# Sex Difference in Cue Strategy in a Modified Version of the Morris Water Task: Correlations between Brain and Behaviour

**DOI:** 10.1371/journal.pone.0069727

**Published:** 2013-07-17

**Authors:** Robin J. Keeley, Amanda V. Tyndall, Gavin A. Scott, Deborah M. Saucier

**Affiliations:** 1 Department of Neuroscience, University of Lethbridge, Lethbridge, Canada; 2 University of Calgary, Calgary, Canada; 3 Faculty of Science, University of Ontario Institute of Technology, Oshawa, Canada; Université Pierre et Marie Curie, France

## Abstract

**Background:**

Sex differences in spatial memory function have been reported with mixed results in the literature, with some studies showing male advantages and others showing no differences. When considering estrus cycle in females, results are mixed at to whether high or low circulating estradiol results in an advantage in spatial navigation tasks. Research involving humans and rodents has demonstrated males preferentially employ Euclidean strategies and utilize geometric cues in order to spatially navigate, whereas females employ landmark strategies and cues in order to spatially navigate.

**Methodology/Principal Findings:**

This study used the water-based snowcone maze in order to assess male and female preference for landmark or geometric cues, with specific emphasis placed on the effects of estrus cycle phase for female rat. Performance and preference for the geometric cue was examined in relation to total hippocampal and hippocampal subregions (CA1&2, CA3 and dentate gyrus) volumes and entorhinal cortex thickness in order to determine the relation between strategy and spatial performance and brain area size. The study revealed that males outperformed females overall during training trials, relied on the geometric cue when the platform was moved and showed significant correlations between entorhinal cortex thickness and spatial memory performance. No gross differences in behavioural performance was observed within females when accounting for cyclicity, and only total hippocampal volume was correlated with performance during the learning trials.

**Conclusions/Significance:**

This study demonstrates the sex-specific use of cues and brain areas in a spatial learning task.

## Introduction

The ability to utilize spatial cues to navigate accurately has demonstrable sex differences. Multiple studies have observed sexually dimorphic spatial learning in rodents [1]–[4] and humans [5]–[7] where males tend to navigate more effectively than females as well as attend to and use different cues for effective navigation. Males preferentially use allocentric cues whereas females preferentially navigate using egocentric cues [3], [8] although females often exhibit greater variability in cue reliance. These findings are recorded in humans, where women used Euclidean-type directions for spatial navigation with less accuracy than men [9].

The hippocampus is tightly linked to spatial memory function, and the volume of the hippocampus and its subregions are sexually dimorphic. Males tend to have a larger right dentate gyri (DG) as compared to females, and females with high circulating estradiol have larger hippocampi as compared to females with low estradiol [10]. Further, increased concentrations of estradiol are associated with increased dendritic spine density in the hippocampus [11] as well as specifically in the CA1 region in rats [12] (for review, see [13]). Further, unilateral lesions of the entorhinal cortex (EC), the main input region to the hippocampus, results in differential responses in males and females, such that males exhibit drastic performance deficits in a spatial memory task whereas females display moderate deficits independent of the size of the lesion [14]. Thus, the role of the both the hippocampus and the EC in spatial learning may be different in males and females.

In a previous study, Rodriguez and colleagues [15] evaluated male and female rats in a modified version of the Morris water task, heretoafter referred to as the snowcone maze, where one quadrant of the pools’ walls met at a 90° angle and the other sides remained circular. The angular shape in the pool served as geometric referent, and a landmark cue was also provided. In this study, males performed better at this task overall, and although males and females were able to process both geometric and landmark cues, males tended to preferentially use the geometric cue whereas females used the landmark cue. Further, using the same paradigm, landmark cues overshadowed geometric cues in females whereas geometric cues overshadowed landmark cues in males [16]. In this series of studies, it was concluded that geometric and landmark cues had different saliency dependent on sex, such that females attended to and took more spatial information from landmark cues and males from geometric cues. However, neither study by Rodriguez and colleagues [15], [17] examined the neural basis of these differences.

As noted above, there are changes in hippocampal morphology across the estrous cycle [10]–[12], [18]. There are also changes in the accuracy of spatial abilities [19] as well as the use of response- or placed-based strategies over the course of the estrous or menstrual cycle [20]. For instance, in a T-maze, female rats with high estrogen will preferentially adopt a place strategy, low estrogen females will preferentially adopt a response strategy and mid estrogen females will show equal numbers adopting both place and response strategies [21]. These studies highlight that circulating concentrations of female sex hormones correlate with spatial learning strategies [22], [23]. Indeed, differential reliance on the hippocampus across estrous phases may account for changes in performance and preference for geometric cues [24]. However, the variability in results present in the literature is large, with some studies reporting improved spatial strategies and performance with high estradiol [25] and others with low [26], [27], and some studies report no difference amongst females related to phase of cycle [28]. Indeed, Rodriguez et al. [15] reported no effect of cycle on performance in the snowcone maze. However, their study used very small sample sizes and manipulated the task to examine how the two cue types interacted with each other rather than a direct examination of the effect of estrous phase *per se*.

Thus, the present study sought to examine sexual dimorphisms in male and female rat preference and performance in the snowcone maze and to correlate behavioural measures with hippocampal subregion and EC volumes following the behavioural task. This task was chosen over other spatial learning tasks such as the standard Morris water task or the Ziggurat maze in order to replicate previous findings as well as examine the effect of cyclicity on female spatial navigation. It was hypothesized that males would outperform females, showing a bias towards the geometric cue, and this increased performance would correlate with greater hippocampal cell densities. For all groups, the thickness of the EC was examined given its function as a main input area to the hippocampus and to examine its role in spatial learning in males and females and the relationship between EC thickness and spatial memory performance. As well, given the variability in the literature, we also investigated how the estrous cycle affects performance in the snowcone maze and its relation to changes in hippocampal and EC morphology. We hypothesized that high estrogen females would preferentially use geometric strategies, and this would correlate with improved performance and greater volumes in all subregions of the hippocampus.

## Materials & Methods

### Ethics Statement

All procedures were approved and conducted in accordance to the University of Lethbridge Animal Welfare Committee and the Canadian Council on Animal Care guidelines.

### Subjects & Handling

Adult Long-Evans rats (10 male and 31 female; ∼250–300 g) were purchased from Charles River (Canada). All rats were housed pairs or triplets in standard shoebox Plexiglas cages (46 cm×25 cm×20 cm) with standard beta-cob bedding. All rats had *ad libitum* access to food and water. Colony rooms were maintained on a 12∶12 light/dark cycle with an average ambient temperature of 21°C and 35% relative humidity. Rats were allowed to adjust to the colony room for 2 weeks following shipping.

### Vaginal Cytology

Phase in estrus cycle was determined using the lavage technique [29]. All samples were taken at approximately the same time of day every day for each rat. A sterile cotton swab was dipped in sterile distilled water and inserted into the vaginal orifice of the female rat and rotated quickly. Once removed, the cotton swab was smeared onto a labeled glass slide (one sample per day per rat). Samples were taken 10–12 days leading up to behavioural testing and on all behavioural testing days. One female rat showed aberrant cyclicity and was excluded from all analyses. Male rats were handled similarly to females, with the cotton swab being used to wipe the scrotum.

### Snowcone Maze

#### Apparatus

**Figure 1:**
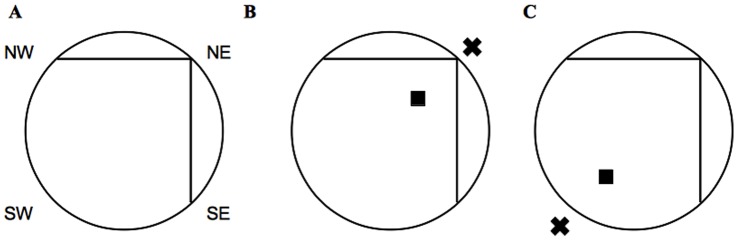
Description of snowcone maze. A. Overheard view of snow cone maze. NE, NW, SW, SE indicate starting positions used on all training days. B. Day 1–3. C. Day 4. Black square indicates platform position. X indicates landmark cue (balloon).

A circular plastic pool (1.5 m diameter, 0.5 m deep) with corrugated plastic inserts (1.09 m long) was filled with 20°C water to a depth of 0.3 m. The corrugated inserts rendered the pool to be of a “snow cone” shape, and the square corner formed by the plastic inserts acted as a geometric cue (for an overhead view of the pool, see Fig. 1a). Water was rendered opaque through the addition of white non-toxic paint (Crayola, USA). Posters of different sizes, shapes and colours hung on the wall of the testing room as well as the computer, rat holding cages and the experimenter served as distal cues. The pool was located roughly 2 m from the N wall, roughly 1.5 m from the E wall, roughly 1.5 m from the S wall and 2 m from the W wall. An overheard video camera attached to a computer (HVS Image, CA, USA) was used to track the rat during all trials. A square (10×10 cm) black Pexiglas platform was placed in the pool 1 cm above the surface of the water and approximately 25 cm form the pool edge. An opaque coloured balloon measuring roughly 20 cm in diameter was hung 15 cm above the platform and was considered a landmark cue.

#### Procedure

All swim trials including probes reached a maximum of 120s, as have been conducted in previous Morris water research [30]. On training days, rats were assigned start positions (NE, NW, SE, SW) in a quasi-random fashion, such that every rat started from all four starting positions at least once each day. The NE starting position was used at a lower frequency in comparison to the other starting locations. On probes days, rats were assigned to start at either the NW or SE starting positions in a quasi-random fashion. On days 1–3, rats were allowed 120s to reach the platform located in the NE quadrant, as marked by the landmark cue (see Fig. 1B). If the rat did not find the platform within that time, they were manually guided to the platform by the experimenter. Once a rat had reached the platform, it would remain there for 15s before being removed and placed in a holding cage until the next trial. The inter-trial interval lasted at least 60s. All rats were given 5 trials every day. On the 4^th^ day of training, the platform and landmark cue (balloon) were moved to the SW quadrant (see Fig. 1C). All rats were given 5 trials. One hour (1 hr probe) and 24 hours (24 hr probe) following the 5^th^ trial on the 4^th^ day, the platform was removed and rats were placed in the pool and allowed to freely swim for 120s.

For all trials, using HVS Image 2020 Plus tracking system (HVS Image Ltd, Bukingham, UK), latency and path length were recorded. For probe trials, percentage of time spend in the NE (area = 0.59 m^2^) or SW (area = 0.78 m^2^) quadrant was recorded as well as heading angle. Heading was quantified, using HVs Image 2020 Plus tracking system, with no bias equaling zero and a heading directed towards the landmark having a negative value and a heading directed toward the geometric cue having a positive value.

### Histology

Rats were euthanized roughly 45 minutes following the 24 hr probe trial. Rats were injected intraperitoneally with sodium pentobarbital (100 mg/kg) and transcardially perfused with approximately 200 mL 0.1 M phosphate-buffered saline (PBS) followed by approximately 200 mL 4% paraformaldehyde in 0.1 M PBS. Rats were decapitated, and brains were extracted and placed in 4% paraformaldehyde overnight then replaced with 30% sucrose with 0.2% Na azide for at least three days before sectioning.

Brains were sectioned on a frozen microtome (Leica, Germany) at a thickness of 40 µm in a series of 12. Sections were stored in 2 mL centrifuge tubes containing 0.1 M PBS and 0.2% sodium azide. One series from each rat was mounted onto 1% gelatin 0.2% chromium-albumin coated glass slides, stained with thionin and cover-slipped with permount.

### Stereology

Estimates of granule cell layer volumes of dentate gyrus (DG), CA1, CA2 and CA3 were conducted using Stereo Investigator software (MicroBrightField Bioscience Inc., USA) and Zeiss Imager M2 microscope (Zeiss, USA). CA1 and CA2 were quantified together due to challenges in delineating one from the other. Cavalieri estimator technique was implemented to estimate volumes using a grid spacing of 100 µm^2^ and a corrected shape factor of 25. Gunderson coefficients of error (m = 0) were consistently below 0.17 for all structures. Due to the vast structural differences in the CA3 region, the coefficient of error was on average higher for this area.

Total HPC volume was quantified using the Cavalieri point-counting method [31]. A Nikon Eclipse 80i microscope with a 1600×1200 megapixel digital colour camera was used to examine each section at a magnification of 2×. Using, Stereologer 2000 software (Stereology Resource Center Inc., Maryland) on a Del Precision computer (T3500) with a live feed from the microscope, a sampling grid with an area per point of 0.2 mm^2^ was randomly superimposed on each section. Grid points were counted if they hit any part of the HPC including all principle cell fields (CA1–3, dentate gyrus, fasciolara cinereum) as well as hippocampal white matter including the oriens layer and alveus. HPC volume was computed by multiplying the total number of points counted for each brain by the area per point and the distance between sections.

## Results

### Behavioural Analyses – Snowcone Maze

#### Latency

**Figure 2:**
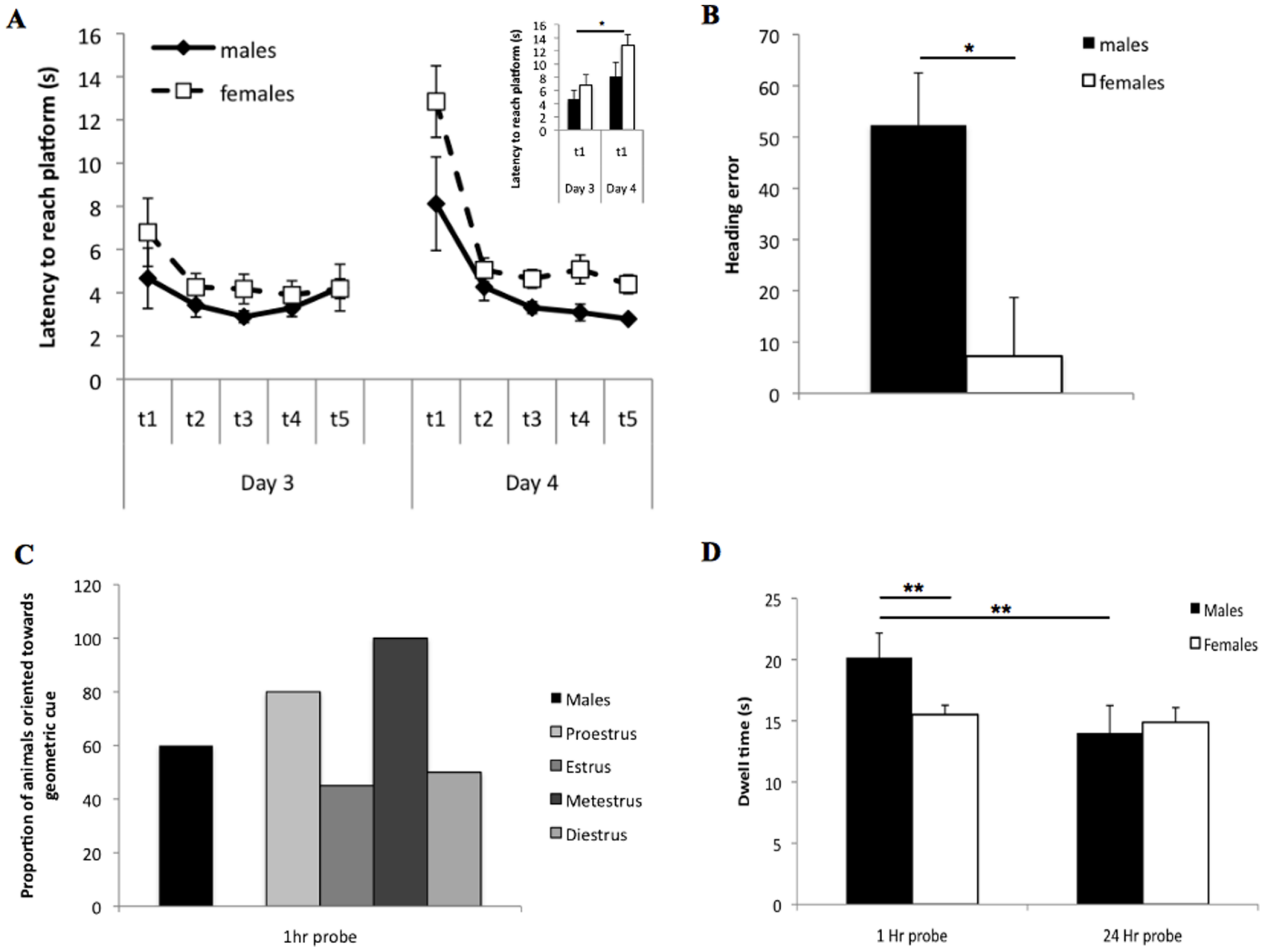
Snowcone maze behaviour. A. Latency to reach platform on day 3 and 4 for males and females. Inset: latency to reach platform on trial 1 for Day 3 and 4. B. Heading error on Day 4 trial 1 for males and females. C. Dwell time in the geometric cue quadrant for males and females. Note the geometric quadrant would be considered the NE quadrant from Fig. 1A. *indicates p<0.05, **indicates p<0.01.

To confirm previous results [15], a 2×5×2 repeated analysis of variance (ANOVA; SPSS version 19) was conducted with day (day 3, day 4) and trial (1–5) as within subjects measures and sex as a between subjects measure was conducted on the latency to reach the platform. ANOVA revealed a main effect of sex, F(1,38) = 5.521, p = .024, a main effect of day, F(1,38) = 7.583, p = .009, and a main effect of trial, F(4,152) = 9.861, p<.001. The main effects were as predicted, with males having shorter latencies than females. However, the main effects of day and trial were mediated by a significant interaction between day and trial, F(4,156) = 3.126, p = .017 (Fig. 2A). There were no other significant effects observed. For the interaction, to reduce the total number of comparisons that were possible, only relevant comparisons, such as Trial 1 of Day 3 compared to Trial 1 of Day 4, were made (Tukey’s HSD). Posthoc analyses indicated that Trial 1 of Day 4 had a significantly longer latency than Trial 1 of Day 3, p<.05. For Day 3, only Trial 1 was significantly longer than the other 4 trials, p<.05, which did not significantly differ among themselves, p values all >.10 (Figure 2A inset). For Day 4, only Trial 1 was significantly longer than the other 4 trials, p<.05, which did not significantly differ among themselves, p values all >.10 (Figure 2A).

Because female rats changed phase of cycle daily and because trial 1 was the only trial for which a significant simple main effect of trial difference was observed, latency to find the platform for Trial 1 on Day 3 was subjected to an ANOVA with group determined on Day 3 (estrus: n = 4; metestrus: n = 10; proestrus: n = 11; diestrus: n = 5; males: n = 10) as a between subjects variable. No significant differences were observed, F(4,35) = 0.756, p = .561. Similarly the latency to find the platform on Trial 1 on Day 4 also failed to reveal significant differences among the groups, as determined on Day 4 (estrus: n = 11; metestrus: n = 4; proestrus: n = 5; diestrus: n = 10; males: n = 10), F(1,36) = .874, p = .489 (data not shown).

#### Path length

To confirm previous results [15], a 2×5×2 repeated analysis of variance was conducted with day (day 3, day 4) and trial (1–5) as within subjects measures and sex as a between subjects measure was conducted on the length of the path taken reach the platform (path length). ANOVA revealed a main effect of sex, F(1,38) = 9.129, p = .004, a main effect of day, F(1,38) = 24.115, p<.001, and a main effect of trial, F(4,152) = 6.859, p<.001. The main effects were as predicted, with males having shorter path lengths than females. However, the main effects of day and trial were mediated by a significant interaction between day and trial, F(4,152) = 5.683, p<.001. There were no other significant effects observed. For the interaction, to reduce the total number of comparisons that were possible, only the relevant comparisons such as Trial 1 of Day 3 compared to Trial 1 of Day 4 were made (Tukey’s HSD). Posthoc analyses indicated that among trials on Day 3, there were no significant differences. Both Trial 1 and Trial 3 were significantly longer on Day 4 than Day 3, p<.05. No other differences reached significance.

Because the female rats changed phase of cycle daily and because Trial 1 was the trial for which the largest significant simple main effect of trial was observed, path length for Trial 1 on Day 3 was subjected to an ANOVA with group (group composition as above) as a between subjects variable. Although it approached significance, this analysis failed to reveal a significant difference among the groups, F(4,35) = 2.445, p = .065. Similarly, the latency to find the platform on Trial 1 on Day 4 failed to reveal significant differences among the groups (group composition as above), F(1,35) = 0.729, p = .578.

#### Heading error

For Day 3, all three cues were in agreement: geometry, landmark and the platform. Thus, heading error was only analyzed for Day 4, Trial 1 and the probe trials, trials in which the landmark was in the opposite quadrant from the geometric cue.

Heading was quantified with no bias equaling zero and a heading directed towards the landmark having a negative value and a heading directed toward the geometric cue having a positive value. For Day 4 Trial 1, ANOVA revealed a significant sex difference, with males having significantly larger positive mean heading angles as compared to females, F(1,38) = 4.434, p = .042 (Fig. 2B). For the 1 hr probe trial, ANOVA failed to observe a sex difference in heading angle scores, F(1,38) = 0.270, p = .606 (data not shown). The same lack of significant sex difference was observed for the 24 hr probe trial, F(1,38) = 471 p = .497 (data not shown).

Given the large variance in heading angle, these values were recoded as directed towards either the geometric cue or landmark cue and subjected to a X^2^ analysis. For Day 4 Trial 1, there was a significant sex difference in the frequency of preference for landmark vs. geometric cues, X^2^(1) = 4.444, p = .035 (Table 1). For the 1 hr probe trial, no significant sex difference in the frequency of preference for landmark vs. geometric cues was observed, X^2^(1) = 0.127, p = .816 (Table 1; see Fig. 2C for a graphical representation). For the 24 hr probe trial, no significant sex difference in the frequency of preference for landmark vs. geometric cues was observed, X^2^(1) = 0.135, p = .714 (Table 1). Although not amenable to analyses due to small samples for some groups, Table 1 also breaks the heading error down by group (estrus, metestrus, proestrus, diestrus, males).

**Table 1:** Heading error.

	Day 4 Trial 1	Probe: 1 hr	Probe: 24 hr
Males	100 (10)	60 (10)	50 (10)
Females	77 (30)	60 (30)	43 (30)
Metestrus	25(4)	100 (4)	45 (11)
Diestrus	70 (10)	50 (10)	25 (4)
Proestrus	60 (5)	80 (5)	60 (10)
Estrus	78 (11)	45 (11)	20 (5)

The number represents the percentage per group that had an initial heading error that indicated initial orientation to the geometric cue (N per group).

#### Probe trials: Time spent in quadrant containing geometric cue

A 2×2 repeated measures ANOVA, with probe trial (1 hr, 24 hr) as a within subjects measure and sex as a between subjects measure, observed a main effect of trial, F(1,38) = 6.087, p = .018, such that rats spent more time in the geometric quadrant during the 1 hr probe trial as compared to the 24 hr probe trial. Howver, there was no main effect of sex, F(1,38) = 1.399, p = .244. There was a significant interaction between sex and trial, F(1,38) = 4.055, p = .043 (Figure 2). Posthoc analyses of simple main effects (Bonferroni) indicated that for the 1 hr probe trial, males spent significantly more time in the quadrant containing the geometric cue than females, p<.01 (Fig. 2D). As well, males spent significantly more time in the quadrant containing the geometric cue during the 1 hr probe trial than the 24 hr probe trial, p<.01. No other significant differences were observed.

Because female rats changed phase of cycle daily, time spent in the quadrant containing the geometric cue for the 1 hr probe trial was subjected to an ANOVA with group (group composition as above) as a between subjects variable and failed to reveal a significant difference among the groups, F(4,35) = 2.061, p = .107. Similarly, the 24 hr probe trial failed to reveal significant differences among the groups (group composition as above), F(4,35) = 0.604, p = .663.

### Histological Analyses

ANOVA revealed that the overall volume of the hippocampus for males was not significantly different than that of females, F(1,38) = 0.007, p = .934. Despite the lack of effect of phase of estrus on behavior, a separate ANOVA performed with group (composition as above) as the between subjects variable revealed a significant difference among the groups, F(4,35) = 3.420, p = .018. Posthoc analyses (Bonferroni) revealed that females at metestrus had significantly larger volumes than either males, p<.01, females at diestrus, p<.001, or estrus, p<.01. No other significant differences were observed among the groups, all p values >.05.

ANOVA revealed that the volume of the DG for males was significantly larger than that of females, F(1,38) = 12.009, p = .001. Despite the lack of effect of phase of estrus on behavior, a separate ANOVA performed with group (composition as above) as the between subjects variable revealed a significant difference among the groups, F(4,35) = 4.024, p = .009. Posthoc analyses (Bonferroni) revealed that males had significantly larger volumes than either females at proestrus or estrus (p<0.05). No other significant differences were observed among the groups.

ANOVA revealed that the volume of CA3 was significantly different among the groups, F(4,35) = 3.394, p = .019 but not between the sexes, F(1,38) = 2.456, p = .125. Posthoc analyses (Bonferroni) revealed that males had significantly larger volumes than females at proestrus.

ANOVA revealed that the combined volume of CA1 and CA2 was not significantly different among the groups, F(4,35) = 1.719, p = .168, nor between the sexes, F(1,38) = 1.060, p = .310.

The maximum thickness of the EC was measured and averaged for the right and left sides. For the maximum thickness, a 2×2 ANOVA with hemisphere (left, right) and sex revealed that the left hemisphere was significantly thicker than the right, F(1,37) = 8.788, p = .005. No other differences reached significance. A 2×5 ANOVA with hemisphere (left, right) and group revealed that there was a main effect of effect of group, F(4,34) = 3.110, p = .028. Posthoc analyses (Bonferroni) revealed that the thickness was greatest for rats in estrus than all other groups, p<.05, except for the rats in diestrus, p>.05 (Table 1). No other differences reached significance (Table 2).

**Table 2:** Maximum entorhinal cortex thickness (mm).

	Hemisphere		Average of RT/LT
	RT	LT	
Males	.00468 (.00017)	.00474 (.00021)	.00471 (.00018)
Females	.00471 (.00025)	.00479 (.00028)	.00475 (.00025)
Metestrus	.00468 (.00023)	.00477 (.00029)	.00472 (.00024)
Diestrus	.00477 (.00021)	.00484 (.00019)	.00481 (.00020)
Proestrus	.00459 (.00022)	.00465 (.00025)	.00462 (.00023)
Estrus	.00499 (.00017)	.00506 (.00021)	.00502 (.00019)

### Correlations between Histology and Behavior

To reduce the total number of possible correlations between the behavioural measures and histological ones, only behavioural data that showed a main effect or interaction with sex were considered (Table 3).

**Table 3:** Correlations.

	AV EC	LT EC	RT EC	HP	DG	CA1&2	CA3
**M path**	.126	.201	.047	.**425** *	.092	.206	.060
**M latency**	−.007	.058	−.069	.**407** *	.242	.088	.168
**D4 heading T1**	−.018	.050	−.088	−.157	.021	.086	.047
**1** **hr probe**	.049	.038	.062	−.142	−.162	−.031	.162
**1** **hr probe** a	.112	.103	.117	−.187	−.159	−.144	.150
**24** **hr probe**	−.249	−.258	−.255	−.072	.006	−.167	.112
**24** **hr probe** a	−.255	−.230	−.174	−.128	.131	.002	−.192

* indicates p<.05, bold indicates that the measure accounts for more than 10% of the variance.

a Spearmans r, with dichotomized variable.

Given that there were sex differences for behavior, separate correlations were completed for males and females (regardless of phase, which failed to reveal behavioural differences; Table 4, Table 5).

**Table 4:** Correlations between behaviour and volume/thickness measurements in male rats.

Males (N = 10)							
	AV EC	LT EC	RT EC	HP	DG	CA1&2	CA3
**M path**	.**400**	.281	.**508** *	.**346**	.046	.213	.058
**M latency**	.284	.136	.**444**	**.639** *	.204	.**408**	−.070
**D4 heading T1**	.**517** *	.**484**	.**507** *	**.551** *	.222	.072	.203
**1** **hr probe**	.**363**	.264	.**448**	−**.367**	−.249	−.054	−.064
**1** **hr probe** a	n.a.**						
**24** **hr probe**	−.**486**	−.**408**	.**544** *	−.193	−.001	−.197	−.054
**24** **hr probe** a	−.**383**	−.313	.**522** *	−.152	−.104	−.313	−.035

* indicates p<.05, bold indicates that the measure accounts for more than 10% of the variance.

a Spearmans r, with dichotomized variable.

** n.a. as all rats headed to the geometric cue.

**Table 5:** Correlations between behaviour and volume/thickness measurements in female rats.

Females (N = 30)								
	AV EC	LT EC	RT EC	HP	DG	CA1&2	CA3	
**M path**	.049	−.035	.137	.**483** *	.**453** *	.141	.218	
**M Latency**	.098	.002	.196	.**417** *	.264	.086	.067	
**D4 heading T1**	−.048	.034	−.133	−.206	−.216	.159	−.068	
**1** **hr probe**	−.006	−.006	.000	−.130	−.123	−.045	.232	
**1** **hr probe** a	.059	.076	.051	−.169	−.134	−.106	.197	
**24** **hr probe**	−.135	−.178	−.084	−.061	.151	−.012	−.260	
**24** **hrprobe** a	−.176	.201	−.084	−.174	.183	.054	−.276	

* indicates p<.05, bold indicates that the measure accounts for more than 10% of the variance.

a Spearmans r, with dichotomized variable.

***Abbreviations:***

CA1–Cornu Ammonis area 1 (hippocampal subregion).

CA2–Cornu Ammonis area 2 (hippocampal subregion).

CA3–Cornu Ammonis area 3 (hippocampal subregion).

DG–dentate gyrus.

EC–entorhinal cortex; note AV EC, LT EC and RT EC correspond to average, left and right entorhinal cortex respectively.

HP–hippocampus.

## Discussion

### Males Outperform Females and are Biased Towards the Geometric Cue

On the third day of training and on the fourth day of training when the platform had changed locations, all rats performed equally well, regardless of sex or phase of estrus for either path length or latency. These results suggest all rats learned the task and that when the platform was present, all rats used this cue to effectively escape the pool, regardless of sex. However, over the course of all training days, there was a main effect of sex such that males reached the platform sooner and traveled a shorter distance to get there than females overall. These results have been observed previously in other water maze tasks [32]–[34], as well as a lack of difference within females accounting for cyclicity for both latency and distance traveled to reach the platform in the snowcone maze [15].

Sex differences emerge when examining heading angles on day 4, with males exhibiting increased accuracy for initial heading. When males were first placed on the pool on day 4, males demonstrated a more accurate heading angle directed towards the geometric cue as compared to females. These results have been paralleled in an earlier study in humans, where initial heading was more accurate in men as opposed to women [35]. Further, the preference for the geometric cue suggests that the geometric cue was a profound influence for males and that females were more variable. The male bias towards the geometric cue is further supported from the increased dwell time in the geometric quadrant for males in the 1 hr probe trial.

Although both sexes demonstrated an overall bias towards the geometric cue, upon further analysis, females in metestrus did not favour the geometric cue on the fourth day of training, with a greater proportion of metestrus females heading towards the landmark cue. This may in fact be an effect of low estrogen during metestrus. However, this is unlikely the case as both metestrus and diestrus females demonstrate similarly low levels of estrogen, and diestrus females performed similarly to other groups. However, higher estrogen groups (proestrus and estrus) demonstrated a large proportion directed towards the geometric cue. Similar results have been observed in high estrogen females demonstrating a preference towards a place strategy in a T-maze [21]. Further research has shown that males are biased towards using a place strategy in the T-maze [36], and this effect is mediated in part by testosterone [37]. Although place strategies and a geometric bias are not the same, parallels can be drawn between the results achieved here regarding bias and previously research examining strategies.

After a day of training and in the absence of the visible platform, neither sex was biased to either cue. Indeed, given both the 1 hr and 24 hr probe, in the repeated absence of a platform, males shifted their strategies, no longer swimming directly towards the geometric cue. However, metestrus females, who did not demonstrate a preference towards the geometric cue on the first trial of the fourth day, shifted their strategies in the absence of a platform in the 1 hr probe, with 100% of the metestrus females directed towards the geometric cue. Perhaps in the absence of a platform, low estradiol females (i.e. females in metestrus) were biased towards a geometric cue. Further, proestrus females also preferentially headed towards the geometric cue, whereas estrus and diestrus females demonstrated a split in their bias. These data are difficult to interpret, as proestrus females would be expected to show the most divergent behaviour due to high levels of circulating sex hormones as compared to all other groups.

Although the interpretation of these results remains difficult, it is clear that fewer females demonstrated a preference towards the geometric cue when the landmark cue was moved. Further, this difference was also expressed across the estrous cycle. Although no clear effect of circulating estrogens is supported from these results, a future study with larger groups numbers may help correlate behavioural results achieved in this experimental paradigm with circulating estrogens in order to support the theory of high circulating estrogens mediating hippocampal overshadowing through cholinergic and N-methyl-D-aspartic acdi-dependent mechanisms (as reviewed in [24]). Indeed, future studies would benefit from conducting all experiments in one day and thus during one day of the estrus cycle, controlling for changes over the course of multiple days. The use of other spatial learning tasks, such as the Ziggurat task, that have demonstrable sex differences in spatial navigation [38] could be employed in order to determine if these findings are consistent across paradigms.

### Males and Females have Equal Total Hippocampal Volumes but Differences in Specific Subregions

Males and females had equal hippocampus volumes. When considering cyclicity, metestrus females had larger total hippocampal volumes compared to males and all other females save for proestrus females. This is counter to what is expected as high levels of estradiol in females have been correlated with larger sizes and greater synaptic densities in subregions of the hippocampus [10], [11], [13]. In this case, a phase consistent with low circulating estradiol was correlated with increased hippocampal volume. Thionin stains cell nuclei, therefore differences in volumes observed here only account for number of cells, not synaptic densities which may account for some of the differences between these results and earlier experiments. Further, the abovementioned experiments used different quantification techniques for their volume estimates. Our results were achieved using non-biased stereology, which has been heralded as the gold standard for volumetric analyses [39]. Therefore, the results achieved here represent a more reliable and unbiased measure as compared to the abovementioned experiments.

Males had overall increases in the DG, with significantly larger DG volumes in comparison to higher estradiol females (proestrus and estrus). This finding differs from a previous study [10], which demonstrated that increased estradiol levels were correlated with increased DG width. However, for males, the present study agrees with the result that males had larger DG as compared to females overall. Discrepancies between this study and the present results may be due to the way in which the DG was examined. The present study compared overall DG volume using non-biased stereology whereas Galea and colleagues quantified right DG width. The quantification method used as well as the precise area of DG quantified may explain any discrepancies. Males also demonstrated increased CA3 volume as compared to proestrus females, which represents a continuation of the results observed in the DG. With high circulating estradiol (proestrus), the DG has a smaller volume, resulting in decreased input to CA3, which also has a lower volume. However, these results did not extend the length of the tri-synaptic circuit as the CA1 showed no differences between groups. Proestrus females did not differ from any other females, indicating that circulating estradiol did not have an effect on overall volume in the DG, CA3 or CA1. Changes in volumes could account for changes in functionality over the course of the estrus cycle, although there was a lack of correlation between volumes of subregions of the hippocampus and any behavioural measure observed. Thus, high estradiol females did not outperform low estrdiol females, nor did high estradiol females demonstrate a bias towards the geometric cue or greater hippocampal cell densities. The lack of differences observed within females for hippocampal subregion volumes and behavioural measures further demonstrated a lack of effect of cyclicity on brain or behavior.

### Total Hippocampal Volume was Correlated with Male and Female Performance only during the Training Trials and not During Probe Trials

The only significant correlation observed between the size of any brain area examined and female spatial navigation performance was a link between latency and path length during the training trials and not during the probe trials. The hippocampus has a well-research role in spatial navigation [40], therefore this relationship is expected. The hippocampus is responsible for the formation of new memories, therefore the significant correlation between the total volume of this structure and performance during training, whether it be latency or path length, is expected and consistent with the current literature.

### Entorhinal Cortex Thickness is Highly Correlated with Male Behavioural Performance

Only the EC showed any observable correlative pattern between volume and spatial memory function, such that both the average and right EC thickness was positively correlated with heading error on the first trial of day 4, and both measures had a negative relationship with the performance on the 24 hr probe. EC function is linked with spatial memory [41], and the present results may provide insight into the precise function of the EC in spatial navigation. The results observed here suggest a predominantly male relationship between EC size and spatial behaviour whereas no such relationship exists in females, indicating other structures may subsume this function in females. Earlier reports support a predominant role of EC in male but not female spatial learning such that unilateral EC lesions produce spatial learning deficits in males but not in females [14]. These observed differences may imply differential functionality of the EC in males and females in relation to spatial memory processing. This theory runs in parallel to other studies that have demonstrated differential activation of brain areas in males and females in response to a spatial task [34], [42]–[45], suggesting sex-specific activation of the brain and reliance on differential neural structures in response to the same spatial task.

## Conclusions

This study is the first, to the author’s knowledge, to demonstrate a significant correlation between all measures of snowcone maze performance and EC volume only in males. Given that this was observed only in males and not females is highly significant as it suggests a sexual divergence of the reliance on EC for geometric cue-biased spatial navigation as both sexes preferentially used this strategy in this study. Differential reliance and links between the EC and spatial memory function between males and females may account for differences in the use of egocentric or allocentric cues, such that males and females rely on and use different neural structures in order to spatially navigate which accounts for the attention and use of differential envivonmental cues. Male predominantly used geometric cues to navigate while females were more variable. This increased variability in the probe trial for the use of geometric cues in females was also accompanied by a lack of significant correlation between performance in the probe trial and hippocampal subfields and entorhinal cortex. This study also has implications on the evolutionary basis of sex differences in spatial memory, such that it demonstrates a potential biological basis for differences in spatial memory and function. Further, the lack of striking differences within females when cyclicity was taken in to account furthers the need to examine whether spatial memory differences are entirely reliant on estradiol status or if other hormonal and biological factors need to be considered.
